# Acute pancreatitis complicated with deep vein thrombosis and pulmonary embolism: a case report

**DOI:** 10.1186/s13256-016-0968-6

**Published:** 2016-06-23

**Authors:** H. M. M. T. B. Herath, Aruna Kulatunga

**Affiliations:** National Hospital Of Sri Lanka, Colombo, Sri Lanka

**Keywords:** Acute pancreatitis, Deep vein thrombosis, Pulmonary embolism, Case report

## Abstract

**Background:**

Acute pancreatitis is an acute inflammatory process of the pancreas that can trigger a systemic inflammatory response. Pulmonary embolism refers to obstruction of the pulmonary artery or one of its branches by material (usually a thrombus) that originated elsewhere in the body. Extensive lower limb deep vein thrombosis with pulmonary embolism is a rare complication of acute pancreatitis that has been described in a few case reports. Deep vein thrombosis and hypercoagulable states in pancreatitis are thought to be due to release of pancreatic proteolytic enzymes from a cyst that is connected to the pancreatic duct and penetrates into a vessel. Proteolytic damage or inflammation of the vessels may also play a significant part. Acute pancreatitis also causes a systemic inflammatory response that has effects on an endothelium-dependent relaxing response for acetylcholine.

**Case presentation:**

A 38-year-old Sri Lankan man presented with acute pancreatitis and later he developed progressive abdominal distention with bilateral ankle edema. A contrast-enhanced computed tomographic scan showed two pancreatic pseudocysts and deep vein thrombosis in both lower limbs, as well as a pulmonary embolism involving the right lower lobe pulmonary artery and the left segmental pulmonary arteries. One of the pseudocysts in the head of the pancreas was compressing the inferior vena cava without direct communication. The patient’s thrombophilia screen result was negative. He was started on subcutaneous enoxaparin 1 mg/kg twice daily and warfarin to achieve a target international normalized ratio of 2–3.

**Conclusions:**

Deep vein thrombosis with pulmonary embolism is a rare but life-threatening complication of acute pancreatitis. Once diagnosed, early treatment with intravenous heparin or thrombolysis is effective. Patients with severe acute pancreatitis may be at risk of deep vein thrombosis due to immobilization and other mechanisms, but anticoagulation as prophylaxis is often not used. However, it may be considered on a case-by-case basis in patients with pancreatitis who are acutely ill and immobilized, need intensive care unit admission, and have multiple risk factors for deep vein thromboembolism. Further studies must be undertaken to determine guidelines for deep vein thromboembolism prophylaxis in these patients.

## Background

Acute pancreatitis is an acute inflammatory process of the pancreas characterized by local tissue injury that can trigger a systemic inflammatory response and is mostly associated with alcoholism or gallstones. The disease is of varying severity. Hemorrhage resulting from an arterial erosion or pseudoaneurysm formation, ischemic complications, and a hypercoagulable state causing venous thrombosis (specifically splanchnic thrombosis) are some of the vascular complications of acute pancreatitis [1].

*Pulmonary embolism* refers to obstruction of the pulmonary artery or one of its branches by material (usually a thrombus) that originated elsewhere in the body. It arises mostly from the deep venous system of the lower extremities [2]. Extensive lower limb deep vein thrombosis (DVT) with pulmonary embolism is a rare complication of acute pancreatitis. In this report, we describe a patient who presented with acute pancreatitis due to chronic alcohol ingestion complicated by bilateral common iliac and left external iliac thrombosis and pulmonary embolism. A pseudocyst at the head of the pancreas was found to be compressing the inferior vena cava. The results of the patient’s genetic and other thrombophilia screens were negative.

## Case presentation

One year before his current presentation, a 38-year-old Sri Lankan man who had previously been well went to the local hospital with intermittent severe epigastric pain radiating to his back, in addition to vomiting. At this initial presentation, acute pancreatitis with high amylase levels was diagnosed. He had been consuming around 12 units of alcohol per week for a 12-year period. He did not have diabetes, hypertension, cholelithiasis, thromboembolic disease, or any other medical disease. He had no family history of significant medical illness. Following this presentation, he had intermittent episodes of epigastric pain, which subsided spontaneously without any medical treatment. At his current presentation, he had a 1-month history of steatorrhea and abdominal pain. His abdominal pain was intermittent and was associated with vomiting. He did not have fever. His initial serum amylase level was high. He was not immobilized. On the third day of admission, he developed progressive abdominal distention with bilateral ankle edema and normal urine output. He was not breathless.

His physical examination revealed that his body mass index was 21.6 kg/m^2^. He was afebrile and pale and had ankle edema. He had a pulse rate of 80 beats per minute, and his blood pressure was 130/80 mmHg. His jugular venous pressure was elevated (8 cm). He was not tachypneic, and both lower zones of his lungs were dull to percussion. His breath sounds were reduced without any added sounds. His abdomen was tensely distended and tender, with gross ascites.

The patient’s amylase level was rising. On day 1 of admission (1 month after symptoms started), his amylase level was 1331 U/L; on day 2, it was 1780 U/L; and on day 3, it was 3570 U/L. His amylase level remained elevated for 3 weeks. His white blood cell count (WBC) was 11.04 × 10^3^ /μL with 70 % neutrophils. His hemoglobin level was 6.7 g/dl, with a hematocrit of 24.9 % on admission (normal 37–54 %). On day 2 of admission, his hematocrit was 25.6 %. His platelet count was 243 × 10^9^/L. Hypochromic microcytic red blood cells (RBC) with a few pencil-shaped cells and macrocytes as well as hypersegmented neutrophils were visualized by mircroscopy. The patient’s reticulocyte index was normal, and the result of his Coombs test was negative. His serum ferritin level was 37.0 μg/L (normal 25–240 μg/L), his serum iron level was 13.4 μg/dl (normal 37–148 μg/dl), his total iron-binding capacity was 296 μg/dl (normal 274–385 μg/dl), and his iron saturation was 4.5 % (normal 15–50 %).

The patient’s liver function tests were within normal range, except for a marginally low albumin level (aspartate aminotransferase 14 U/L, alanine transaminase 10 U/L, alkaline phosphatase 125 U/L, total bilirubin 18 μmol/L, total protein 48 g/L, albumin 32 g/L, globulin 16 g/L, international normalized ratio [INR] 1.23). His erythrocyte sedimentation rate was 56 mm/h in the first hour, and his C-reactive protein (CRP) levels were 35 mg/L on admission and 24 mg/L (normal 0–5 mg/L) after 48 hours. His serum ionized calcium on day 2 of admission was 0.91 mmol/L (normal 1.09–1.3 mmol/L), and it was 1.14 mmol/L with replacement of calcium on day 5. His serum creatinine concentration was within normal range throughout (75, 88, and 78 μmol/L; normal 60–120 μmol/L : on days 1,2 and 5 respectively), and his blood urea nitrogen level was normal (2.6 mmol/L, normal 2.9–8.2 mmol/L). His serum sodium level was 133 mmol/L, and his serum potassium concentration was 3.3 mmol/L. His random blood sugar level on admission was 126 mg/dl. Arterial blood gas analysis showed a pH of 7.5 with partial pressure of carbon dioxide of 31.4 mmHg, bicarbonate of 30.0 mmol/L, base excess of +8.0, lactate of 1.0 mmol/L (normal 1.0–2.5 mmol/L), oxygen saturation of 95.8 %, and partial pressure of oxygen of 73.4 mmHg. His fasting blood sugar level was 5.6 mmol/L (normal <5.6 mmol/L). His lipid profile was normal, with a normal triglyceride level of 120 mg/dl. His thyroid-stimulating hormone level was 0.97 mIU/L (normal 0.55–4.78 mIU/L), and his free thyroxine was 1.54 (normal 0.89–1.76).

**Fig. 1:**
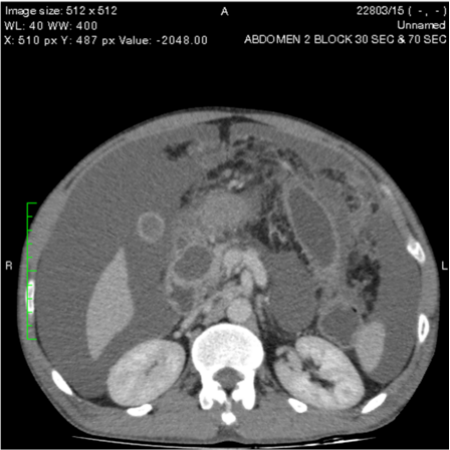
Two pancreatic pseudocysts, measuring 4.5 cm × 3.5 cm and 4.2 cm × 5 cm, respectively, in relation to the pancreatic head

An ultrasound scan of the abdomen showed gross ascites with a normal liver and kidneys. Contrast-enhanced computed tomography (CECT) of the abdomen revealed two pancreatic pseudocysts, measuring 4.5 cm × 3.5 cm and 4.2 cm × 5 cm, respectively, in relation to the pancreatic head (Fig. 1). The pancreatic duct and the rest of the pancreas were normal, without evidence of necrosis or changes of chronic pancreatitis. Gross ascites was visualized on both an ultrasound scan of the abdomen and a CECT scan. The patient’s bowel appeared normal. His peritoneal fluid was an exudate with 4.3 g/dl protein and a serum-to-ascites albumin gradient of −1.1 g/dl (<1.1 g/dl), a lactic acid dehydrogenase level of 527 U/L, WBC of 73 cells/mm^3^ with 90 % lymphocytes, and RBC of 1.6 × 10^9^/mm^3^. His ascitic fluid amylase level was high at 3618 IU/L. No malignant cells were seen, and the patient’s Gram stain and acid-fast bacilli smear results were negative, with the culture remaining sterile. His adenosine deaminase level was normal (12 IU/L).

**Fig. 2:**
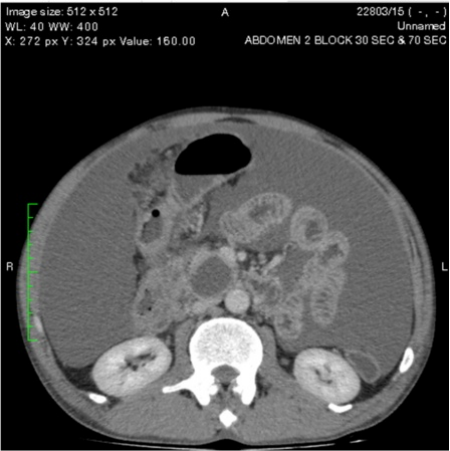
One of the pseudocysts in the head of the pancreas compressing the inferior vena cava

**Fig. 3:**
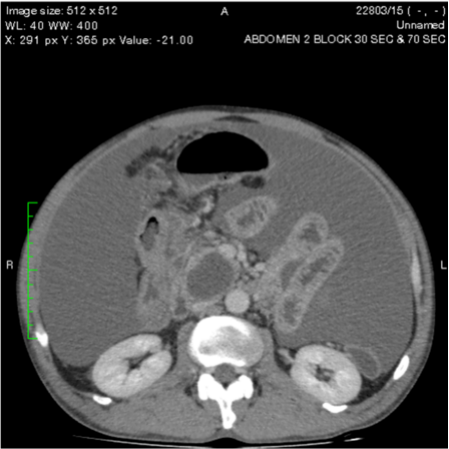
The pseudocysts compressing the inferior vena cava without any direct communication

**Fig. 4:**
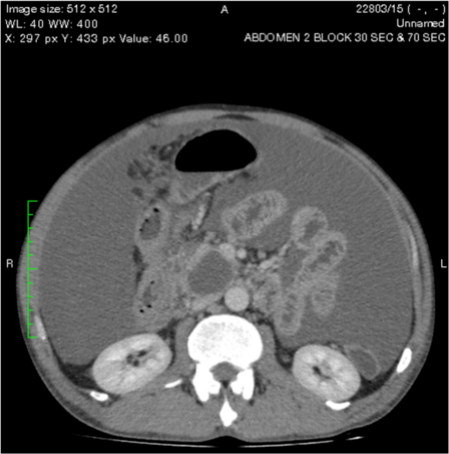
The level that the inferior vena cava maximally got compressed

**Fig. 5:**
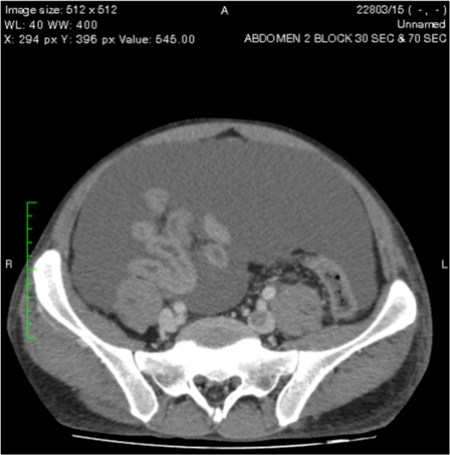
Filling defects in the left common iliac vein

**Fig. 6:**
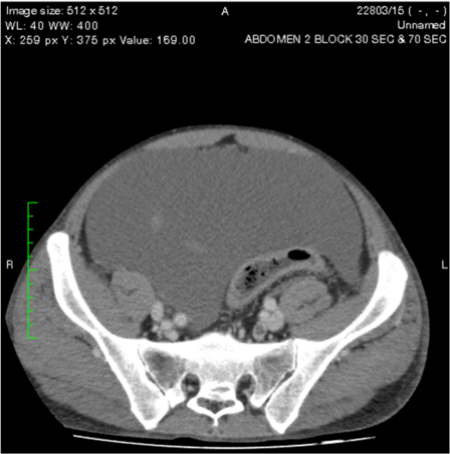
Filling defects in the left internal iliac vein

**Fig. 7:**
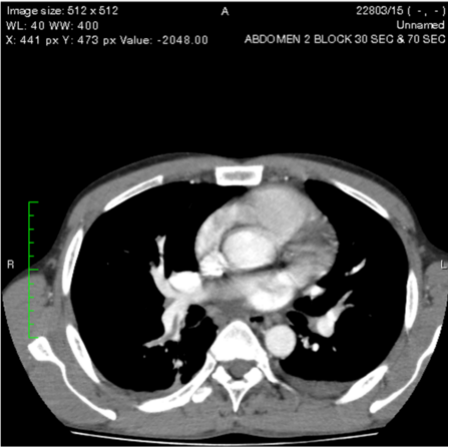
Multiple filling defects were seen in the right lower lobe pulmonary artery

**Fig. 8:**
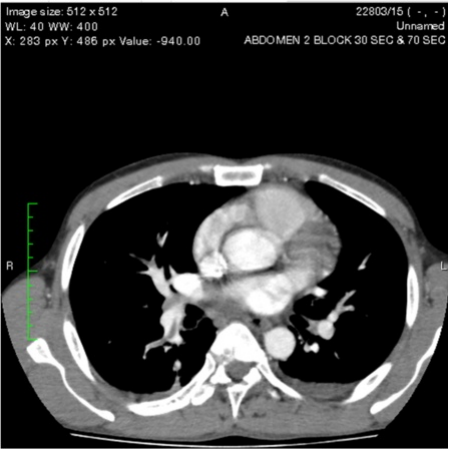
Multiple filling defects were seen in the right lower lobe pulmonary artery

One of the pseudocysts in the head of the pancreas was compressing the inferior vena cava (IVC), as shown in the CECT scans in Figs. 2, 3 and 4. There were filling defects in the left common and internal iliac veins and both proximal external iliac veins, suggesting DVT (Figs. 5 and 6). The patient’s IVC was patent. Multiple filling defects were seen in the right lower lobe pulmonary artery and in segmental branches of the left pulmonary artery, compatible with pulmonary embolism (Figs. 7 and 8). The patient’s liver, gallbladder, spleen, kidneys, and adrenal glands were normal. He had bilateral atelectasis of the lung bases with minimal pleural effusions.

Venous Doppler sonography of the patient’s lower limbs did not reveal DVT in the femoral and popliteal veins. The patient’s D-dimer level was 1.43 mg/L. His left ventricular ejection fraction was 55 %, with diastolic dysfunction visualized on a two-dimensional echocardiogram. His main pulmonary artery was normal, with a pressure gradient of 23 mmHg, and his right ventricular function was good.

His antinuclear antibody and anticardiolipin antibody test results were negative. He had no nocturnal hematuria, and the findings in three consecutive early morning samples were negative for hemosiderin. The results of genetic testing for prothrombin gene mutation, factor V Leiden, and *MTHFR* gene mutation were negative. The results of the patient’s Ham test and the thrombophilia screen for antithrombin III as well as protein C and protein S deficiency were negative. His test result for dengue antibodies was negative. The results of his monospot test for Epstein-Barr virus, hepatitis B surface antigen, and hepatitis C antibodies were negative. His test result for HIV was also negative, and his VDRL was nonreactive. His upper gastrointestinal endoscopy was normal initially. His carcinoembryonic antigen level was 0.9 μg/L (normal <5 μg/L).

After the diagnosis of DVT and pulmonary embolism was made, the patient was started on subcutaneous enoxaparin 1 mg/kg twice daily and warfarin to achieve a target INR of 2–3. Initially, he was kept nil orally; later, gradual enteral feeding was introduced. Calcium was replaced orally. Later, he developed bleeding into the peritoneal cavity with a high INR, and 6 U of blood were transfused. He was managed in the intensive care unit (ICU) during this period, and total parenteral nutrition was given. His bleeding settled spontaneously with INR correction. His ascitic fluid and large pseudocyst were drained using a pigtail catheter. The pigtail catheter was kept in place until the drainage stopped, and then it was removed. After 10 days in the ICU, the patient recovered and was discharged on oral warfarin to achieve a target INR of 2–3. He was planned to be treated with anticoagulation for 6 months because he did not have any other acquired or congenital risk factors for thromboembolism.

## Discussion

Acute pancreatitis is inflammation of the pancreas that presents as abdominal pain with elevated levels of pancreatic enzymes in the blood. Serum amylase is the most frequent test used to diagnose acute pancreatitis, and computed tomography is the most important imaging tool for the diagnosis and assessment of severity of pancreatitis.

**Fig. 9:**
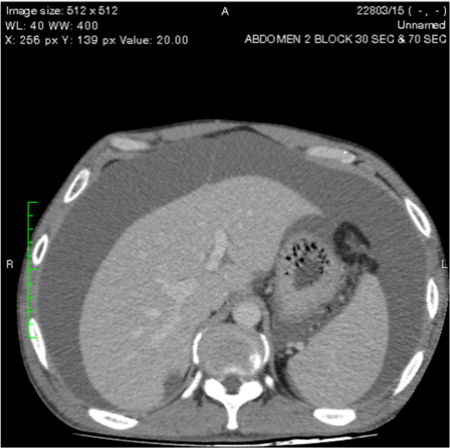
Gross ascites at the level of the liver and spleen

**Fig. 10:**
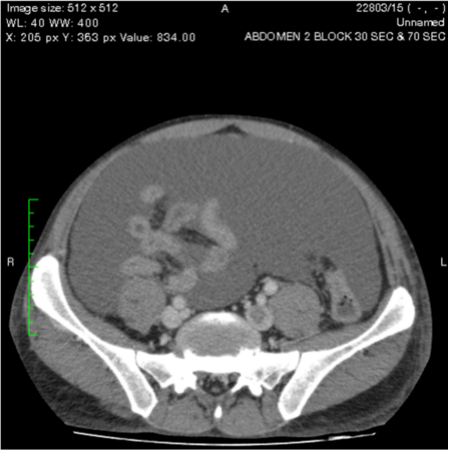
Gross ascites with freely floating bowel loops

According to the revised Atlanta classification system, our patient had interstitial edematous acute pancreatitis, a disease in which there is acute inflammation of the pancreatic parenchyma and peripancreatic tissues but without tissue necrosis [3]. The patient had no hemoconcentration, and his CRP was below 150 mg/dl at 48 hours [4]. His Balthazar score was grade 4 [5]. His Ranson criteria scores were 0 on admission and 2 at 2 hours, giving a 0–3 % mortality [6]. His Acute Physiology and Chronic Health Evaluation II score was 8.0 points with 6 % estimated nonoperative mortality [4]. His Bedside Index for Severity in Acute Pancreatitis score was 1, with a mortality rate less than 2 %. His gross ascites was disproportionate to the severity of his pancreatitis, but this could be explained by the presence of a pancreatic fistula producing pancreatic ascites (Figs. 9 and 10).

Vascular complications of pancreatitis are a major cause of morbidity and mortality. Venous complications are predominantly related to splanchnic vein thrombosis due to local inflammation [1]. Pulmonary embolism is a rare complication of pancreatitis that has been described in few case reports [7]. Renal vein and IVC thrombosis associated with acute pancreatitis has been reported [8]. DVT and hypercoagulable states in pancreatitis are thought to be due to release of pancreatic proteolytic enzymes from a cyst that is connected to the pancreatic duct and penetrates into a vessel. The pancreatic juice triggers the formation of a thrombus secondary to vasculitis. Proteolytic damage or inflammation of the vessels may also play a significant part, and pancreatic elastase has been shown to play a major role in the development of pulmonary vascular injury after acute pancreatitis [9]. It is also thought that hypercoagulability in pancreatitis is due to a combination of hepatic dysfunction and hypertrypsinemia (resulting in raised fibrinogen and factor VIII concentrations) and cachexia. Acute pancreatitis provokes deleterious effects in the endothelium-dependent relaxing response for acetylcholine in mesenteric rings that are strongly associated with high plasma nitrite/nitrate levels as a consequence of intense inflammatory responses. Furthermore, the subsensitivity of the contractile response to phenylephrine in both mesenteric and pulmonary rings might be due to the complications of this pathological condition in the early stage of pancreatitis [10]. In DVT, immobility may also contribute. In our patient, one of the pancreatic pseudocysts was compressing the IVC, as shown on CECT of the abdomen, although direct communication was not seen.

A thrombophilic state leading to venous thrombosis can be inherited or acquired. Our patient did not have any acquired causes and had negative test results for other prothrombotic conditions. Therefore, we conclude that the patient’s DVT and pulmonary embolism were secondary to acute pancreatitis.

Early recognition, as well as investigation and diagnosis, of pulmonary embolisms is important, because early treatment with intravenous heparin and radiological interventional procedures such as vascular filters can reduce mortality. The American College of Chest Physicians 2008 guidelines [11] recommend venous thromboembolism prophylaxis with low-molecular-weight heparin in patients undergoing major general surgery, major gynecologic surgery, major open urologic procedures, and elective hip or knee arthroplasty, as well as in all major trauma and spinal cord injury patients and patients admitted to the hospital with an acute medical illness. On admission to the ICU, all patients should be assessed for their risk of venous thromboembolism, and most should receive thromboprophylaxis.

## Conclusions

Deep vein thrombosis with a pulmonary embolism is a rare but life-threatening complication of acute pancreatitis. If clinically suspected, necessary investigations should be arranged. Once diagnosed, early treatment with intravenous heparin or thrombolysis is effective, and other causes of thrombophilia should be looked for. Patients with severe acute pancreatitis may be at risk of DVT due to immobilization and the mechanisms mentioned above in the Discussion section. However, anticoagulation as prophylaxis is often not used, because these patients may need intervention (pigtail catheter drainage or surgery) and may be at an increased risk of bleeding [12]. It may be considered on a case-by-case basis in patients with pancreatitis who are acutely ill, immobilized, need ICU admission, and have multiple risk factors for deep vein thromboembolism. Further studies must be undertaken to determine guidelines for deep vein thromboembolism prophylaxis in these patients.
